# Building Meaningful Relationships for Equity in the Publishing Ecosystem: Empowering Latin American Research Through Engagement

**DOI:** 10.1002/ece3.71964

**Published:** 2025-09-01

**Authors:** Bruno E. Soares, Victória P. Barbosa, Naraiana L. Benone, Castiele H. Bezerra, Lucas F. Colares, Marília M. S. Costa, Vicente V. Faria, José A. M. Fernandes, Guilherme Gama, Leonora Torres‐Knoop, Camila R. O. Leal, Romullo G. Lima, Raiana Lima, Guilherme Maricato, Francisco T. V. Melo, Mariana M. Moreira‐Lima, Bianca Nandyara, Rita de C. Q. Portela, Bruno S. Prudente, Jonathan S. Ready, Carla Rezende, Luciana L. Santos, Amanda S. Santos, Samuel Washington, Ana C. B. Siqueira, Alexandre S. Siqueira, Welber S. Smith, Rodrigo Tardin, Gustavo C. Tavares, Bruno Umbelino, Gustavo Vancellote, Lorenzo R. S. Zanette, Arley Muth

**Affiliations:** ^1^ Institute of Environmental Change & Society University of Regina Regina Saskatchewan Canada; ^2^ Programa de Pós‐Graduação em Ecologia Universidade Federal do Rio de Janeiro Rio de Janeiro Brazil; ^3^ Programa de Pós Graduação em Ecologia e Evolução Universidade Federal de São Paulo São Paulo Brazil; ^4^ Departamento de Biociências Universidade do Estado de Minas Gerais Passos Brazil; ^5^ Programa de Pós‐Graduação em Ecologia e Recursos Naturais Universidade Federal do Ceará Fortaleza Brazil; ^6^ National Institute of Science and Technology in Synthesis of Amazonian Biodiversity Belém Brazil; ^7^ Programa de Pós‐graduação em Sistemática, Uso e Conservação da Biodiversidade – PPGSis Universidade Federal do Ceará Fortaleza Brazil; ^8^ Programa de Pós‐graduação em Engenharia de Pesca – PPGEP Universidade Federal do Ceará Fortaleza Brazil; ^9^ Programas de Pós‐graduação em Ciências Marinhas Tropicais – PPGCMT Universidade Federal do Ceará Fortaleza Brazil; ^10^ Instituto de Ciências Biológicas Universidade Federal do Pará Belém Brazil; ^11^ Programa de Pós‐Graduação Em Biologia de Agentes Infecciosos e Parasitários, Instituto de Ciências Biológicas Universidade Federal do Pará Belém Brazil; ^12^ Instituto Socioambiental e dos Recursos Hídricos Universidade Federal Rural da Amazônia Belém Brazil; ^13^ Programa de Pós‐Graduação em Ecologia e Recursos Naturais Universidade Federal de São Carlos São Carlos Brazil; ^14^ Programa de Pós‐Graduação em Patologia Ambiental e Experimental Universidade Paulista São Paulo Brazil; ^15^ Departamento de Ecologia, Instituto de Biologia Universidade Federal do Rio de Janeiro Rio de Janeiro Brazil; ^16^ John Wiley & Sons Ltd Hoboken New Jersey USA

**Keywords:** capacity‐building, decolonial science, global collaboration, Global South, scientific publishing

## Abstract

Equity in scientific publishing requires removing financial barriers, structural transformation, and inclusive practices that empower researchers from historically marginalized regions. Here, we reflect on recent Wiley's initiatives supporting Brazilian researchers to integrate into the international publishing ecosystem, including discounted rates for open‐access article processing charges, the Wiley‐CAPES transformative agreement, and in‐country capacity‐building events. While some challenges persist, such as linguistic barriers and funding access, we underscore the importance of meaningful local engagement and the coordinated actions among publishers and funding agencies that are supporting a more equitable publishing ecosystem.

## Abstract 2

This Letter to the Editor reflects on recent efforts to promote equity in scientific publishing for Latin American researchers. We are drawing on our collective experiences and perspectives to underscore some persistent challenges and advances in more inclusive, locally engaged publishing practices that support Global South leadership.

## 1 Introduction

Ecological and conservation challenges are inherently global. The drivers and ecological consequences of climate change, biodiversity loss, biological invasions, and other pressing issues transcend national borders; and so must their solutions (Allen et al. 2024; Dukes and Mooney 1999; Svenning et al. 2024). Ecological research and practice must be international in scope and in participation if aiming to effectively develop conservation strategies and understand ecosystem dynamics (Milner‐Gulland 2021). This goal has been advancing through global agreements and assessments (Betts et al. 2020; Hughes and Grumbine 2023), large‐scale efforts for organizing and synthesizing information (Dornelas et al. 2025; Scholes et al. 2012), and international cooperation (Borer et al. 2025; Loescher et al. 2022).

However, deep disparities persist in who publishes, leads global research, and receives recognition from the international community (Miller et al. 2023; Nakamura et al. 2023; Nuñez et al. 2019; Smit et al. 2025). Despite knowledge gaps in biodiversity science (e.g., Carvalho et al. 2023; Nakamura et al. 2025; Vergara‐Asenjo et al. 2023), Latin American (LA) researchers strongly contribute to biodiversity research. An example is the substantial contribution of primary data from LA included in global databases and/or meta‐analyses (e.g., Dornelas et al. 2025; Ferreira et al. 2024; Lima et al. 2025; Poisot et al. 2021). LA researchers face systemic barriers to gain visibility and recognition in global publishing venues, such as peer‐reviewing biases (Fox et al. 2023), language barriers (Amano et al. 2023), and financial barriers (Smith et al. 2021; Turba et al. 2025) emerging from historical and political factors. For example, intellectual production in Latin America faced numerous barriers during periods of authoritarianism, and it remains affected by the fragility of democracy in some countries (Beigel 2014). These factors have reinforced LA's dependence on international publishing circuits that are often misaligned with local research priorities and epistemologies. Addressing such inequities is not only the ethical pathway to dismantle the barriers imposed by colonial histories (Soares et al. 2023) but is also necessary to strengthen the actions underpinning global conservation (Amano et al. 2021).

In this editorial, we highlight some recent initiatives of Wiley—the publisher of *Ecology & Evolution* (E&E)—to support Latin American researchers. These include a pricing parity for open‐access article processing charges, a transformative agreement with Brazil's Coordenação de Aperfeiçoamento de Pessoal de Nível Superior (CAPES), and a series of in‐country workshops connecting editors and researchers organized by editors of E&E, *Journal of Biogeography*, and other Wiley titles. These actions underscore the academic landscape's progress in addressing global research inequities and the importance of aligning policy with local practice to achieve this goal. Furthermore, this editorial amplifies the voices of several participants from the above‐mentioned workshops, representing diverse career levels and regions of Brazil, in conjunction with the voice of a Brazilian researcher working abroad and current leaders in academic publishing from the Global North. Altogether, our perceptions underscore the progress that global science has recently made toward equitable publishing, the pressing need for structural reform in international and national institutions, and the importance of meaningful engagement from Global North publishers operating in the Global South.

## 2 Policy and Partnership Toward Equity

Promoting equity in scientific publishing requires action at multiple levels, from publishers to funding agencies (Arenas‐Castro et al. 2024; Chankseliani 2023). Recently, Wiley has introduced two concrete mechanisms to make publishing openly more attainable for researchers in LA: (i) a pricing parity model that offers discounted open‐access fees considering the gross domestic product of countries; and (ii) a transformative agreement with CAPES, a federal Brazilian agency under Brazil's Ministry of Education that supports higher education, covering the open‐access charges for articles published in hybrid journals. The pricing parity policy, currently implemented as a 1‐year pilot program, offers a discounted rate for publishing in Wiley's gold open‐access journals to authors from LA countries. Brazil, for example, benefits from a 55% reduction in open‐access fees. Several workshop participants recognized this effort as a meaningful step toward reducing inequalities in accessing open publishing. As one of us noted, “These actions are quite effective. I believe open publishing can become more accessible through policies such as this.”

At the same time, pricing parity must be accompanied by stronger institutional support from Brazilian funding agencies to have a lasting impact. The recent transformative agreement between Wiley and CAPES is a step in that direction. This agreement ensures that articles accepted in Wiley's hybrid journals by authors affiliated with Brazil are published under an open‐access license without fees, thereby removing a major financial barrier for Brazilian researchers. In 2024, Brazilian researchers published 55 open‐access articles in Wiley's hybrid journals. As of June 2025, under the terms of this agreement, 563 articles by Brazilian researchers had already been openly published in the same journals. Such an agreement is crucial for reducing inequalities driven by regional disparities in access to research and education funds in Brazil (Guimarães et al. 2020; Yamamoto 2000). To put this into perspective, from 2016 to 2022, the Southeast region of Brazil—which includes states such as São Paulo and Rio de Janeiro—received approximately 40% (about US$360,000) of all biodiversity research funding in the country (Stegmann et al. 2024). In contrast, the North region of Brazil, home to the Amazon rainforest, received only about 10% of that funding (~US$87,000) during the same period. Given these disparities, transformative agreements, such as the Wiley‐CAPES partnership, can play a crucial role in promoting open‐access publishing for researchers in poorly funded regions. Nevertheless, several participants emphasized that greater support from host institutions and funding agencies remains necessary, particularly to enable publishing in fully open‐access (gold) journals not covered by the transformative agreement.

The growing number of policies, such as transformative agreements, institutional publishing contracts, and APC discounts or waivers, is reshaping the publishing landscape and making it more inclusive. These policies facilitate access to the global publishing landscape for Brazilian researchers, demonstrating how multilateral, coordinated action can reshape global access to science while providing mutual benefits to all parties involved through increased visibility and more equitable knowledge exchange (Borrego et al. 2021; Collyer 2018). The CAPES‐Wiley transformative agreement (TA) is just one of these recent policies, and similar agreements can be found between publishers and institutions worldwide. In January 2025, ESAC (https://esac‐initiative.org), which maintains a public record of TAs signed by publishers and diverse stakeholders, recorded over 1 000 agreements in more than 70 countries and more than 300 000 articles published in 2024. These agreements vary in structure and scope; some offer limited discounts or waivers, while others provide unlimited open‐access publishing (Borrego et al. 2021). The long‐term impacts of such policies have yet to be assessed, and it remains critical to ensure that these mechanisms are designed to promote a more equitable distribution of resources and responsibilities across the global research community.

## 3 Equity Goes Beyond Access

While agreements addressing accessibility are essential to advance equity in publishing, they must be complemented by meaningful, on‐the‐ground engagement, because barriers to publishing are not only financial. Participants highlighted challenges such as the lack of institutional guidance, language barriers, and unfamiliarity with publishing systems and norms established elsewhere. Many of us would even describe the process as intimidating, especially for early‐career researchers or those disconnected from highly internationalized urban institutions. Therefore, tackling such challenges should include addressing the cultural and linguistic barriers imposed by the international publishing ecosystem; for example, the expected structure for submitted articles and the dominant norms about what is considered proper academic writing. For many early‐career researchers, especially those trained outside Anglophone institutions from which such norms are emerging, navigating these rules in a second language poses additional struggles and anxiety (Amano et al. 2023). Some journals are responding to such barriers by changing procedural norms, such as allowing free formatting at the initial submission stage, which might diminish the cognitive and emotional burden of engaging with an unfamiliar system.

Language emerges as a central barrier to inclusion in the international publishing ecosystem. English is considered the *lingua franca* in international scientific publishing, thereby increasing the cognitive and financial burden for authors whose English is a Second Language (ESL) and acting as a form of gatekeeping, that is, filtering who gains access to the publishing ecosystem (Barzilai‐Nahon 2009; Habibie and Hultgren 2022). Participants mentioned that previous harsh interactions with reviewers regarding their English led to delays in submission or having to spend scarce resources on costly editing services.

Tackling this barrier demands both support mechanisms and a shift in editorial attitudes toward embracing linguistic diversity. Efforts to break language barriers have been adopted by some journals, such as the option to include second language abstracts or even full‐texts to enhance accessibility in the region where the study was carried out. Take the special issue “Ecology in South America: Present state and future prospects” as an example (Zenni et al. 2024), in which articles were published in Portuguese (Brazil‐Sousa et al. 2024; Cerqueira et al. 2024; Hidasi‐Neto et al. 2024; Ligo et al. 2024) or Spanish (Alvarez Dalinger et al. 2024; López‐Bedoya et al. 2024; Sotomayor et al. 2024; Spirito et al. 2024) in addition to the English version. Recent advances in Artificial Intelligence (AI) can also serve as valuable tools in supporting ESL authors; for example, the use of AI for automatically translating oral presentations in conferences from speakers' first languages (Fair et al. 2024). A suggestion would be for publishers to implement systems that automatically support authors in academic writing when submitting to the journal.

At the same time, publishers should critically assess how their editorial systems and filters are reinforcing colonial epistemologies and ways of doing science (Asase et al. 2022; De Vos and Schwartz 2022), as well as promoting further marginalization of Indigenous, traditional, and other non‐Western forms of knowledge (Santos 2015). Actions such as reserving a portion of the funding from grant calls or setting explicit goals for increasing the participation of Global South researchers as authors, reviewers, and editors would promote higher inclusivity in the publishing ecosystem. For example, the British Ecological Society reserves 51% of the funding in its biannual calls for researchers from the Global South. Another example is a dedicated section in Ecology & Evolution for Indigenous knowledge (McKinnon and Muth 2024), establishing editorial infrastructure to accommodate diverse forms of knowledge and facilitating access to a more interdisciplinary ecology (Meng et al. 2025; Obrist et al. 2024). These actions not only promote inclusion but also epistemological justice, recognizing the importance of historically silenced forms of knowledge for the advancement of global science.

A common approach to breaking these divides has been through internationalization efforts and increased push for collaborations between institutions in the Global North and Global South (Paiva and Brito 2019; Soares et al. 2025). North–South cooperation is often seen as a pathway to build capacity for ecological research and practice in less economically favored countries (Blicharska et al. 2021). However, we argue that true equity demands that such cooperation be guided by Global South leadership—not as passive recipients of an inclusion dictated by the Global North, but as the ones shaping the inclusion of the Global North in our (all authors but KG and AM) research and conservation actions (Ocampo‐Ariza et al. 2023; Soares et al. 2023). It is also necessary to redesign how funding is distributed within collaborative projects with Global South partners, who are often left with minimal resources and no agency in managing funds, thus allowing the Global North to maintain the power to make decisions. Without such changes, even well‐meaning initiatives risk reproducing postcolonial dynamics in which the norms—such as what counts as “equity” or “capacity‐building”—are still externally defined. A decolonial approach to collaboration, by contrast, fosters long‐lasting and trust‐based relationships centering co‐development and local knowledge (Toomey et al. 2019). Therefore, while policy changes and editorial reform are essential, they should be matched by on‐the‐ground relational efforts.

## 4 Equity Is Built on Meaningful Relationships

In the spirit of tackling the above‐mentioned barriers to international publishing ecosystems, a Brazilian researcher based abroad (BES) partnered with Brazil‐based researchers and a publishing Global North company (Wiley) to carry out capacity‐building efforts. We conducted a series of in‐person workshops, entirely funded by Wiley, in March 2025 at four Brazilian universities: Universidade de São Paulo (USP), Universidade Federal do Rio de Janeiro (UFRJ), Universidade Federal do Ceará (UFC), and Universidade Federal do Pará (UFPA). These workshops brought together over 300 participants, including journal editors, publishing professionals, and researchers from different career stages (> 90% early‐career researchers) to share experiences and demystify the publishing process through open dialog. As one participant said, “The most important part for me was having a first direct contact with those on the other side of the publishing process.”

Sessions covered practical aspects of the publishing process, such as understanding open‐access models, navigating journal scopes, preparing strong submissions, responding effectively to peer review, and interpreting publishing agreements. Sessions also explored broader topics such as research visibility, ethical authorship, and how to frame contributions for international readerships. In addition, the workshops were intentionally structured to create space for open dialog and mutual learning, and participants were encouraged to raise specific concerns and discuss their perceptions on barriers they face when publishing. Informal conversations, Q&A sessions, and group reflections allowed participants to share personal experiences with publishing, including frustrations and strategies. Several noted that this was their first direct contact with a journal editor or publishing professional, and that this encounter helped to demystify the image of international editors and emphasized their potential as allies. “I felt safe to ask questions I've been carrying for years,” said one early‐career researcher. “It was the first time I saw editors as people willing to help, not gatekeepers.” The relational dimension of these workshops was particularly powerful: by (mostly) speaking in Portuguese, in their familiar places, participants seemed more empowered to voice their questions and concerns, and to see themselves as part of the global scientific community.

Our efforts were guided by the belief that equity is not achieved through financial policy alone, but through the everyday work of building meaningful relationships. Researchers need more than access to read and publish article; they need mentors, advocates, and opportunities to lead. That leadership must be rooted in local contexts, shaped by those who understand the realities of their communities and institutions, and capable of building bridges across global, national, and local systems. The workshops were an attempt to model this kind of engagement: regionally grounded, dialogic, and mutually accountable. Building on their success, we are exploring ways to expand this approach to remote and hybrid formats, allowing broader participation while retaining the relational foundation that made these first attempts so impactful.

## 5 Conclusion

While we celebrate the progress represented by these recent initiatives—from pricing parity to transformative agreements and capacity‐building initiatives—true equity in publishing still demands deep structural transformation to ensure representation from different racial and ethnic groups, geographic regions, and career stages. Global South researchers must be empowered to lead not only as authors but also as reviewers, editors, and decision‐makers who can also shape the international publishing ecosystem. This includes reassessing exclusionary or biased practices, diversifying editorial boards, and strengthening long‐term capacity‐building (Espin et al. 2017; Naidu et al. 2024; Pettorelli et al. 2021; Primack et al. 2019). In parallel, strengthening South–South partnerships is essential to ensure that collaboration is not only driven by Global North institutions, but also rooted in shared experience, regional leadership, and solidarity across the Global South.

We hope these recent advances are not met with resistance or reversed in the face of shifting political priorities, but instead serve as starting points for a deeper—and sometimes uncomfortable—conversation about what it means to internationalize science in a way that is aligned with global justice. We invite readers to join this conversation, offer feedback, and explore ways to support, replicate, or expand these efforts across other regions and institutions. We should also learn from regions facing similar challenges in their academic landscape—particularly across the Global South, including Latin America, where researchers often face shared socioeconomic and environmental struggles. Equity in publishing requires more than access: it requires reimagining leadership, accountability, recognition, and the systems that define whose voices are heard.

## Author Contributions

**Bruno E. Soares:** conceptualization (equal), writing – original draft (equal). **Victória P. Barbosa:** writing – review and editing (equal). **Naraiana L. Benone:** writing – review and editing (equal). **Castiele H. Bezerra:** writing – review and editing (equal). **Lucas F. Colares:** writing – review and editing (equal). **Marília M. S. Costa:** writing – review and editing (equal). **Vicente V. Faria:** writing – review and editing (equal). **José A. M. Fernandes:** writing – review and editing (equal). **Guilherme Gama:** writing – review and editing (equal). **Leonora Torres‐Knoop:** writing – review and editing (equal). **Camila R. O. Leal:** writing – review and editing (equal). **Romullo G. Lima:** writing – review and editing (equal). **Raiana Lima:** writing – review and editing (equal). **Guilherme Maricato:** writing – review and editing (equal). **Francisco T. V. Melo:** writing – review and editing (equal). **Mariana M. Moreira‐Lima:** writing – review and editing (equal). **Bianca Nandyara:** writing – review and editing (equal). **Rita de C. Q. Portela:** writing – review and editing (equal). **Bruno S. Prudente:** writing – review and editing (equal). **Jonathan S. Ready:** writing – review and editing (equal). **Carla Rezende:** writing – review and editing (equal). **Luciana L. Santos:** writing – review and editing (equal). **Amanda S. Santos:** writing – review and editing (equal). **Ana C. B. Siqueira:** writing – review and editing (equal). **Alexandre S. Siqueira:** writing – review and editing (equal). **Welber S. Smith:** writing – review and editing (equal). **Rodrigo Tardin:** writing – review and editing (equal). **Gustavo C. Tavares:** writing – review and editing (equal). **Bruno Umbelino:** writing – review and editing (equal). **Gustavo Vancellote:** writing – review and editing (equal). **Samuel Washington:** writing – review and editing (equal). **Lorenzo R. S. Zanette:** writing – review and editing (equal). **Arley Muth:** writing – review and editing (equal).

## Conflicts of Interest

The authors declare no conflicts of interest.

## Data Availability

No data were created or analyzed in this manuscript.

## References

[ece371964-bib-0001] Allen, B. J. , D. J. Hill , A. M. Burke , et al. 2024. “Projected Future Climatic Forcing on the Global Distribution of Vegetation Types.” Philosophical Transactions of the Royal Society B: Biological Sciences 379, no. 1902: 20230011. 10.1098/rstb.2023.0011. PMC10999268 38583474

[ece371964-bib-0002] Alvarez Dalinger, F. S. , V. L. Lozano , L. Moraña , C. N. Borja , and M. Salusso . 2024. “¿Los Índices Biológicos de Calidad Son Universalmente Útiles? Límites de los Índices Basados en Bentos en Una Subcuenca Salobre y Árida de Argentina.” Austral Ecology 49, no. 1: e13340. 10.1111/aec.13340.

[ece371964-bib-0003] Amano, T. , V. Berdejo‐Espinola , A. P. Christie , et al. 2021. “Tapping Into Non‐English‐Language Science for the Conservation of Global Biodiversity.” PLoS Biology 19, no. 10: e3001296. 10.1371/journal.pbio.3001296. 34618803 PMC8496809

[ece371964-bib-0004] Amano, T. , V. Ramírez‐Castañeda , V. Berdejo‐Espinola , et al. 2023. “The Manifold Costs of Being a Non‐Native English Speaker in Science.” PLoS Biology 21, no. 7: e3002184. 10.1371/journal.pbio.3002184. 37463136 PMC10353817

[ece371964-bib-0005] Arenas‐Castro, H. , V. Berdejo‐Espinola , S. Chowdhury , et al. 2024. “Academic Publishing Requires Linguistically Inclusive Policies.” Proceedings of the Royal Society B: Biological Sciences 291, no. 2018: 20232840. 10.1098/rspb.2023.2840. PMC10932714 38471557

[ece371964-bib-0006] Asase, A. , T. I. Mzumara‐Gawa , J. O. Owino , A. T. Peterson , and E. Saupe . 2022. “Replacing “Parachute Science” With “Global Science” in Ecology and Conservation Biology.” Conservation Science and Practice 4, no. 5: e517. 10.1111/csp2.517.

[ece371964-bib-0007] Barzilai‐Nahon, K. 2009. “Gatekeeping: A Critical Review.” Annual Review of Information Science and Technology 43, no. 1: 1–79. 10.1002/aris.2009.1440430117.

[ece371964-bib-0061] Beigel, F. 2014. “Introduction: Current Tensions and Trends in the World Scientific System.” Current Sociology 62, no. 5: 617–625. 10.1177/0011392114548640.

[ece371964-bib-0008] Betts, J. , R. P. Young , C. Hilton‐Taylor , et al. 2020. “A Framework for Evaluating the Impact of the IUCN Red List of Threatened Species.” Conservation Biology 34, no. 3: 632–643. 10.1111/cobi.13454. 31876054 PMC7318271

[ece371964-bib-0009] Blicharska, M. , C. Teutschbein , and R. J. Smithers . 2021. “SDG Partnerships May Perpetuate the Global North–South Divide.” Scientific Reports 11, no. 1: 22092. 10.1038/s41598-021-01534-6. 34824306 PMC8617181

[ece371964-bib-0010] Borer, E. , E. Seabloom , I. Slette , et al. 2025. “Species Cover, Community Biomass, and Richness in Global Grasslands From NutNet (2007–2023): Dominant Species Predict Plant Richness and Biomass in Global Grasslands [Dataset].” Environmental Data Initiative. 10.6073/PASTA/B9DF4CB39A594B1BF0732085B69B74E5.

[ece371964-bib-0011] Borrego, Á. , L. Anglada , and E. Abadal . 2021. “Transformative Agreements: Do They Pave the Way to Open Access?” Learned Publishing 34, no. 2: 216–232. 10.1002/leap.1347.

[ece371964-bib-0012] Brazil‐Sousa, C. , B. E. Soares , R. Svanbäck , and M. P. Albrecht . 2024. “A Especialização Individual é Maior em Populações Generalistas de Posições Tróficas Intermediárias a Altas em Peixes Tropicais de Água Doce.” Austral Ecology 49, no. 1: e13397. 10.1111/aec.13397.

[ece371964-bib-0013] Carvalho, R. L. , A. F. Resende , J. Barlow , et al. 2023. “Pervasive Gaps in Amazonian Ecological Research.” Current Biology 33, no. 16: 3495–3504.e4. 10.1016/j.cub.2023.06.077. 37473761

[ece371964-bib-0014] Cerqueira, A. F. , M. Benchimol , C. Sousa‐Santos , et al. 2024. “Tendências e Lacunas na Literatura Sobre Palmeiras Nativas da Mata Atlântica Brasileira.” Austral Ecology 49, no. 1: e13414. 10.1111/aec.13414.

[ece371964-bib-0015] Chankseliani, M. 2023. “Who Funds the Production of Globally Visible Research in the Global South?” Scientometrics 128, no. 1: 783–801. 10.1007/s11192-022-04583-4.

[ece371964-bib-0016] Collyer, F. M. 2018. “Global Patterns in the Publishing of Academic Knowledge: Global North, Global South.” Current Sociology 66, no. 1: 56–73. 10.1177/0011392116680020.

[ece371964-bib-0017] De Vos, A. , and M. W. Schwartz . 2022. “Confronting Parachute Science in Conservation.” Conservation Science and Practice 4, no. 5: e12681. 10.1111/csp2.12681.

[ece371964-bib-0018] Dornelas, M. , L. H. Antão , A. E. Bates , et al. 2025. “BioTIME 2.0: Expanding and Improving a Database of Biodiversity Time Series.” Global Ecology and Biogeography 34, no. 5: e70003. 10.1111/geb.70003. PMC6099392 30147447

[ece371964-bib-0019] Dukes, J. S. , and H. A. Mooney . 1999. “Does Global Change Increase the Success of Biological Invaders?” Trends in Ecology & Evolution 14, no. 4: 135–139. 10.1016/S0169-5347(98)01554-7. 10322518

[ece371964-bib-0020] Espin, J. , S. Palmas , F. Carrasco‐Rueda , et al. 2017. “A Persistent Lack of International Representation on Editorial Boards in Environmental Biology.” PLoS Biology 15, no. 12: e2002760. 10.1371/journal.pbio.2002760. 29232375 PMC5726619

[ece371964-bib-0021] Fair, H. , O. A. Medina‐Báez , B. J. Spiecker , et al. 2024. “Can AI Interpretation Increase Inclusivity?” Frontiers in Ecology and the Environment 22, no. 10: e2821. 10.1002/fee.2821.

[ece371964-bib-0022] Ferreira, V. M. B. , B. E. Soares , J. S. Leal , and M. P. Albrecht . 2024. “Spatial Factors Overcome Seasonality Regulating the Consumption of Allochthonous Food Resources by Fishes From Tropical Lotic Ecosystems.” Freshwater Biology 69, no. 6: 783–791. 10.1111/fwb.14245.

[ece371964-bib-0023] Fox, C. W. , J. Meyer , and E. Aimé . 2023. “Double‐Blind Peer Review Affects Reviewer Ratings and Editor Decisions at an Ecology Journal.” Functional Ecology 37, no. 5: 1144–1157. 10.1111/1365-2435.14259.

[ece371964-bib-0024] Guimarães, A. R. , C. D. S. Brito , and J. A. B. Dos Santos . 2020. “Expansão e Financiamento da Pós‐Graduação e Desigualdade Regional No Brasil (2002–2018).” Práxis Educacional 16, no. 41: 47–71. 10.22481/praxisedu.v16i41.7244.

[ece371964-bib-0025] Habibie, P. , and A. K. Hultgren . 2022. “Different Faces of Gatekeeping and Gatekeepers.” In The Inner World of Gatekeeping in Scholarly Publication, edited by E. P. Habibie and A. K. Hultgren , 9–24. Springer International Publishing. 10.1007/978-3-031-06519-4_2.

[ece371964-bib-0026] Hidasi‐Neto, J. , N. M. A. Gomes , and N. S. Pinto . 2024. “A Vegetação Nativa do Cerrado é um Refúgio Para as Aves na Trajetória Atual da Mudança Climática.” Austral Ecology 49, no. 1: e13336. 10.1111/aec.13336.

[ece371964-bib-0027] Hughes, A. C. , and R. E. Grumbine . 2023. “The Kunming‐Montreal Global Biodiversity Framework: What It Does and Does Not Do, and How to Improve It.” Frontiers in Environmental Science 11: 1281536. 10.3389/fenvs.2023.1281536.

[ece371964-bib-0028] Ligo, A. B. , R. D. S. Laurindo , L. D. B. Faria , and R. Gregorin . 2024. “Fatores Climáticos, Geográficos e Antropogênicos Moldam a Estrutura das Redes Morcego‐Fruto na Região Neotropical.” Austral Ecology 49, no. 1: e13378. 10.1111/aec.13378.

[ece371964-bib-0029] Lima, R. G. d. S. F. , B. E. Soares , M. Cadotte , and M. P. Albrecht . 2025. “Freshwater Fish Functional Diversity Shows Diverse Responses to Human Activities, but Consistently Declines in the Tropics.” Ecography: e07746. 10.1002/ecog.07746.

[ece371964-bib-0030] Loescher, H. W. , R. Vargas , M. Mirtl , et al. 2022. “Building a Global Ecosystem Research Infrastructure to Address Global Grand Challenges for Macrosystem Ecology.” Earth's Future 10, no. 5: e2020EF001696. 10.1029/2020EF001696.

[ece371964-bib-0031] López‐Bedoya, P. A. , T. Magura , D. M. Méndez‐Rojas , J. A. Noriega , F. G. Horgan , and D. P. Edwards . 2024. “Conocimiento de las Comunidades de Escarabajos Que Habitan el Suelo en los Andes Tropicales: Lagunas y Tendencias.” Austral Ecology 49, no. 1: e13440. 10.1111/aec.13440.

[ece371964-bib-0032] McKinnon, E. A. , and A. F. Muth . 2024. “Introducing a New Special Section—Indigenous Science and Practice in Ecology and Evolution.” Ecology and Evolution 14, no. 7: e11718. 10.1002/ece3.11718. 39055777 PMC11269205

[ece371964-bib-0033] Meng, R. L. , A. McGregor , D. McGregor , L. McGregor , K. Nahwegahbow , and P. Chow‐Fraser . 2025. “A Framework for Doing Things *in a Good Way*: Insights on Mshiikenh (Freshwater Turtle) Conservation Through Weaving Western Science and Indigenous Knowledge in Whitefish River First Nation.” Ecology and Evolution 15, no. 5: e71431. 10.1002/ece3.71431. 40342699 PMC12061448

[ece371964-bib-0034] Miller, J. , T. B. White , and A. P. Christie . 2023. “Parachute Conservation: Investigating Trends in International Research.” Conservation Letters 16, no. 3: e12947. 10.1111/conl.12947.

[ece371964-bib-0035] Milner‐Gulland, E. J. 2021. “The Global Conservation Movement Is Divided but Not Diverse: Reflections on 2020.” Oryx 55, no. 3: 321–322. 10.1017/S003060532100048X.

[ece371964-bib-0036] Naidu, T. , C. Cartmill , S. Swanepoel , and C. R. Whitehead . 2024. “Shapeshifters: Global South Scholars and Their Tensions in Border‐Crossing to Global North Journals.” BMJ Global Health 9, no. 4: e014420. 10.1136/bmjgh-2023-014420. PMC11029397 38724078

[ece371964-bib-0037] Nakamura, G. , L. C. J. Corvalán , L. B. Paula‐Souza , et al. 2025. “Darwinian Shortfall and Macroecological Patterns in Genetic Data of Tocantins‐Araguaia Basin Fishes.” Neotropical Ichthyology 23, no. 1: e240047. 10.1590/1982-0224-2024-0047.

[ece371964-bib-0038] Nakamura, G. , B. E. Soares , V. D. Pillar , J. A. F. Diniz‐Filho , and L. Duarte . 2023. “Three Pathways to Better Recognize the Expertise of Global South Researchers.” Npj Biodiversity 2, no. 1: 17. 10.1038/s44185-023-00021-7. 39242772 PMC11332133

[ece371964-bib-0039] Nuñez, M. A. , J. Barlow , M. Cadotte , et al. 2019. “Assessing the Uneven Global Distribution of Readership, Submissions and Publications in Applied Ecology: Obvious Problems Without Obvious Solutions.” Journal of Applied Ecology 56, no. 1: 4–9. 10.1111/1365-2664.13319.

[ece371964-bib-0040] Obrist, D. S. , E. J. Pendray , R. D. Field , et al. 2024. “Comparing Historical and Contemporary Observations of Avian Fauna on the Yáláƛi (Goose Island) Archipelago, British Columbia, Canada.” Ecology and Evolution 14, no. 12: e70464. 10.1002/ece3.70464. 39633782 PMC11615508

[ece371964-bib-0041] Ocampo‐Ariza, C. , M. Toledo‐Hernández , F. Librán‐Embid , et al. 2023. “Global South Leadership Towards Inclusive Tropical Ecology and Conservation.” Perspectives in Ecology and Conservation 21, no. 1: 17–24. 10.1016/j.pecon.2023.01.002.

[ece371964-bib-0042] Paiva, F. M. , and S. H. A. D. Brito . 2019. “O Papel da Avaliação CAPES No Processo de Internacionalização da Pós‐Graduação em Educação no Brasil (2010–2016).” Avaliação: Revista da Avaliação da Educação Superior (Campinas) 24, no. 2: 493–512. 10.1590/s1414-40772019000200009.

[ece371964-bib-0043] Pettorelli, N. , J. Barlow , M. A. Nuñez , et al. 2021. “How International Journals Can Support Ecology From the Global South.” Journal of Applied Ecology 58, no. 1: 4–8. 10.1111/1365-2664.13815.

[ece371964-bib-0044] Poisot, T. , G. Bergeron , K. Cazelles , et al. 2021. “Global Knowledge Gaps in Species Interaction Networks Data.” Journal of Biogeography 48, no. 7: 1552–1563. 10.1111/jbi.14127.

[ece371964-bib-0045] Primack, R. B. , T. J. Regan , V. Devictor , et al. 2019. “Are Scientific Editors Reliable Gatekeepers of the Publication Process?” Biological Conservation 238: 108232. 10.1016/j.biocon.2019.108232.

[ece371964-bib-0046] Santos, B. D. S. 2015. Epistemologies of the South. Routledge. 10.4324/9781315634876.

[ece371964-bib-0047] Scholes, R. J. , M. Walters , E. Turak , et al. 2012. “Building a Global Observing System for Biodiversity.” Current Opinion in Environmental Sustainability 4, no. 1: 139–146. 10.1016/j.cosust.2011.12.005.

[ece371964-bib-0048] Smit, I. P. J. , R. J. Fernández , M. F. Menvielle , et al. 2025. “From Parachuting to Partnership: Fostering Collaborative Research in Protected Areas.” Journal of Applied Ecology 62, no. 1: 28–40. 10.1111/1365-2664.14814.

[ece371964-bib-0049] Smith, A. C. , L. Merz , J. B. Borden , C. K. Gulick , A. R. Kshirsagar , and E. M. Bruna . 2021. “Assessing the Effect of Article Processing Charges on the Geographic Diversity of Authors Using Elsevier's “Mirror Journal” System.” Quantitative Science Studies 2, no. 4: 1123–1143. 10.1162/qss_a_00157.

[ece371964-bib-0050] Soares, B. E. , A. C. S. Franco , J. S. Leal , R. G. D. S. F. Lima , K. Baker , and M. Griffiths . 2023. “Decolonising Ecological Research: A Generative Discussion Between Global North Geographers and Global South Field Ecologists.” Area 55, no. 4: 550–557. 10.1111/area.12901.

[ece371964-bib-0051] Soares, B. E. , A. L. Moura , V. N. D. Araújo , et al. 2025. “The “Conhecimento Brasil” Program Neglects the Structural Problems of Brazilian Science and Fails to Offer a Solution to the Brain Drain.” Anais da Academia Brasileira de Ciências 97, no. 1: e20240496. 10.1590/0001-3765202520240496. 40105591

[ece371964-bib-0052] Sotomayor, D. A. , C. Caro , and R. Morales . 2024. “Una Revisión Sistemática de las Tendencias en Ciencia Ecológica en el Perú Megabiodiverso: Vacíos en Investigación y Orientaciones Futuras.” Austral Ecology 49, no. 1: e13371. 10.1111/aec.13371.

[ece371964-bib-0053] Spirito, F. , P. Meli , M. F. Reyes , G. Núñez‐Vivanco , Z. Beloff , and J. L. De Paepe . 2024. “Estereotipos de Género en los Temas de Investigación en Ecología: Un Análisis de los Últimos 20 Años de las Reuniones Argentinas de Ecología.” Austral Ecology 49, no. 1: e13372. 10.1111/aec.13372.

[ece371964-bib-0054] Stegmann, L. F. , F. M. França , R. L. Carvalho , et al. 2024. “Brazilian Public Funding for Biodiversity Research in the Amazon.” Perspectives in Ecology and Conservation 22, no. 1: 1–7. 10.1016/j.pecon.2024.01.003.

[ece371964-bib-0055] Svenning, J.‐C. , M. A. McGeoch , S. Normand , A. Ordonez , and F. Riede . 2024. “Navigating Ecological Novelty Towards Planetary Stewardship: Challenges and Opportunities in Biodiversity Dynamics in a Transforming Biosphere.” Philosophical Transactions of the Royal Society B: Biological Sciences 379, no. 1902: 20230008. 10.1098/rstb.2023.0008. PMC10999270 38583480

[ece371964-bib-0056] Toomey, A. H. , M. E. C. Alvaro , M. Aiello‐Lammens , O. Loayza Cossio , and J. Barlow . 2019. “A Question of Dissemination: Assessing the Practices and Implications of Research in Tropical Landscapes.” Ambio 48, no. 1: 35–47. 10.1007/s13280-018-1056-5. 29691805 PMC6297105

[ece371964-bib-0057] Turba, R. , E. S. J. Thoré , M. G. Bertram , et al. 2025. Global North‐South Science Inequalities due to Language and Funding Barriers. Zenodo. 10.5281/ZENODO.14902147.

[ece371964-bib-0058] Vergara‐Asenjo, G. , F. M. Alfaro , and J. Pizarro‐Araya . 2023. “Linnean and Wallacean Shortfalls in the Knowledge of Arthropod Species in Chile: Challenges and Implications for Regional Conservation.” Biological Conservation 281: 110027. 10.1016/j.biocon.2023.110027.

[ece371964-bib-0059] Yamamoto, O. H. 2000. “Financiamento da Pesquisa no Brasil: Distorções e Desigualdades.” Estudos de Psicologia (Natal) 5, no. 2: 279–287. 10.1590/S1413-294X2000000200001.

[ece371964-bib-0060] Zenni, R. D. , A. Andersen , N. R. Andrew , et al. 2024. “Ecology in South America: Present State and Future Prospects.” Austral Ecology 49, no. 1: e13457. 10.1111/aec.13457.

